# D-Chiro-Inositol in Endometrial Hyperplasia: A Pilot Study

**DOI:** 10.3390/ijms241210080

**Published:** 2023-06-13

**Authors:** Giuseppina Porcaro, Gabriele Bilotta, Elena Capoccia, Maria Salomé Bezerra Espinola, Cesare Aragona

**Affiliations:** 1 Women’s Health Centre, USL Umbria 2, 05100 Terni, Italy; 2 The Experts Group on Inositol in Basic and Clinical Research (EGOI), 00156 Rome, Italy; espinolasalome@gmail.com (M.S.B.E.); aragonacesare@gmail.com (C.A.); 3 Alma Res Fertility Center, 00198 Rome, Italy; gabriele.bilotta90@gmail.com (G.B.); elena.capoccia753@gmail.com (E.C.); 4 Systems Biology Group Lab, 00161 Rome, Italy

**Keywords:** D-chiro-inositol, endometrial hyperplasia, unopposed estrogens, abnormal uterine bleeding, heavy menstrual bleeding, aromatase

## Abstract

Endometrial hyperplasia is a threatening pathology driven by unopposed estrogen stimulus. Moreover, insulin may act on the endometrium, prompting further growth. We aimed at assessing whether D-chiro-Inositol, an insulin sensitizer with estrogen-lowering properties, might improve the condition of patients with simple endometrial hyperplasia without atypia. We enrolled women with simple endometrial hyperplasia without atypia and related symptoms, including abnormal uterine bleeding. We treated the patients with one tablet per day, containing 600 mg of D-chiro-inositol for six months. Patients underwent ultrasound to assess the thickness of the endometrium at baseline, after three months, and at the end of this study. Endometrial thickness went from 10.82 ± 1.15 mm to 8.00 ± 0.81 mm after three months (*p* < 0.001) and to 6.9 ± 1.06 mm after six months (*p* < 0.001 versus baseline; *p* < 0.001 versus three months). D-chiro-inositol treatment also improved heavy menstrual bleeding and the length of menstruation. Despite the fact that our data should be validated in larger studies with appropriate control groups, our promising results support the hypothesis that D-chiro-inositol may represent a useful treatment in the case of endometrial hyperplasia without atypia.

## 1. Introduction

Perimenopause is the moment in the life of women that marks the transition from fertile life to climacteric. One of the first events that distinguish this period is an ovarian failure, which causes an increase in Follicle-Stimulating Hormone (FSH) levels to stimulate estrogen production and ovulation [1]. In this condition, women may face a condition of deregulated production of estrogens. If, on the one hand, the ovaries may suffer from insensitivity to FSH, leading to a reduced response to stimulation and a scarce production of estrogens, on the other hand, the gonads may respond to such overstimulation [2]. In such conditions, estrogen production is triggered to a pathologically great extent, allowing the excess estrogens to induce changes in the physiological milieu of the uterus. The condition of high estrogen is known as hyperestrogenism [3]. Hyperestrogenism belongs to a wider range of disturbances called hormonal imbalances.

Such excess estrogens may be balanced by progesterone levels, as progesterone acts in an opposite manner from estrogen, giving rise to contrasting phenomena. Indeed, estrogen and progestogen have different receptors and different intracellular pathways, but their physiological and clinical outcomes are nearly opposite. The results of the excess estrogens compared to progesterone levels may cause hyperproliferative phenomena. In fact, estrogens induce the proliferation of reproductive tissues, while progesterone accounts for differentiative processes [4,5]. Therefore, the balance between estrogen and progesterone is crucial for avoiding potentially harmful hormonal stimulation. A disbalance between estrogen and progesterone levels occurs both with high estrogen levels coupled with normal progesterone levels or with normal estrogen levels coupled with low progesterone levels. In clinical terms, this phenomenon is known as “unopposed estrogens”, and it represents a risk factor for several estrogen-induced pathologies [3]. The gynecological conditions related to unopposed estrogen are characterized by the overexpression of aromatase, the enzyme that converts the androgens into estrogens [6]. Aromatase is the only enzyme that can produce estrogens in the human body; therefore, its expression and activity represent the key point in the development of unopposed estrogens. Indeed, aromatase is overexpressed in the pathologies induced by unopposed estrogens, as it provides pivotal factors for the development and for the progression of such pathologies [3,4,7].

Unopposed estrogens can induce abnormal uterine bleeding (AUB), which is usually the predominant symptom. Among the phenomena that go under the name of AUB, menorrhagia or heavy menstrual bleeding (HMB) is one of the most burdensome and is generally associated with elevated endometrial thickness [2]. In addition, unusually long menses represent a form of AUB, and, therefore, physicians can monitor the length of the periods and the days of heavy bleeding. During perimenopause, other symptoms related to sexual life may occur due to the physiological reduction in testosterone levels, including vaginal distress and reduction in sexual desire [8]. Besides unopposed estrogens, insulin may stimulate cell proliferation as well. Therefore, hyperinsulinemia represents a risk factor for several pathologies characterized by excessive cell growth, including estrogen-sensible pathologies. Indeed, different studies revealed that metabolic abnormalities, such as obesity, type 2 diabetes, hypertension, and dyslipidemia, represent risk factors for endometrial hyperplasia or endometrial cancer [9,10].

In order to improve unopposed-estrogen-related symptomatology, physicians usually prescribe hormonal treatments, mainly progestogens, which have non-negligible side effects [11]. In this regard, clinicians should consider that aging is highly related to an increased risk of adipose tissue growth, hypertension, and diabetes. For these reasons, the use of hormonal therapies must be carefully evaluated in women who have preexisting risk factors [1]. Another important factor to consider is the length of therapy, especially in reproductive-age patients, as progesterone-based treatments are contraceptive drugs. In addition to hormonal treatments, recent research focused on the use of insulin sensitizers to reduce the growth stimulus in patients with hyperinsulinemia or insulin resistance [12]. These studies showed promising results, allowing the researchers to speculate that insulin sensitizers may also be helpful in the case of hormonal treatments. Indeed, some clinical trials combined standard progestogen-based therapies with insulin sensitizers, such as metformin, reporting intriguing results [10,13]. Moreover, several recent trials tested whether the class of drugs known as aromatase inhibitors has an effect in the case of unopposed estrogens and related pathologies [14,15]. Indeed, aromatase inhibitors are drugs designed to fill the active site of the aromatase, preventing the functioning of the enzyme. As a result, the aromatase produced in the body loses its ability to catalyze reactions, thus reducing the synthesis of estrogens. Intriguingly, the trials with aromatase inhibitors in women suffering from unopposed estrogens report promising results in the symptomatology [14,15].

Considering the estrogen- and insulin-dependent mechanisms of growth involved in proliferative pathologies, recent research focused on the possibility of reducing both stimuli [10,16]. In this regard, the recent literature reported the potential effect of D-chiro-inositol (DCI), a natural molecule with insulin-sensitizing effects widely described in the literature. DCI is commonly used in clinical practice as an insulin sensitizer in different pathologies, such as Polycystic Ovary Syndrome (PCOS). Nonetheless, the recent literature suggests that the use of such molecules in this clinical picture should be carefully evaluated [17,18,19,20,21,22]. Indeed, recent publications highlighted that DCI has an effect of transcriptional inhibition on aromatase, allowing the speculation that DCI may reduce the excess of estrogens [3,23]. This effect could lead to restoring the correct hormonal balance in patients suffering from endometrial thickening and/or AUB, especially in the case of hyperestrogenism. Additionally, there is evidence of minor or null side effects following DCI treatment, contrary to what happens with progestogens, which are known to have relevant adverse effects [16,17]. Therefore, our pilot study aimed at evaluating the efficacy of D-chiro-inositol in treating women with endometrial thickening and related symptoms as AUB.

## 2. Results

Patients attending the gynecological department with typical symptoms of pathologies characterized by unopposed estrogens (HMB and long menstruations) were screened for inclusion and exclusion criteria. Among twenty patients screened for this study, thirteen fulfilled the inclusion criteria and matched no exclusion criteria. Anthropometric data belonging to patients are reported in Table 1. The patients had no other medical comorbidities associated with the endometrial thickening.

All thirteen women enrolled had heavy menstrual bleeding episodes, and such episodes lasted for longer than 3 days for twelve of them. At the baseline, the mean value of endometrial thickness measured on the 10th day of the menstrual cycle was equal to 10.8 mm, associated with menses lasting on average for 8.8 days, of whom 5.3 days, on average, were characterized by heavy bleeding.

**Figure 1:**
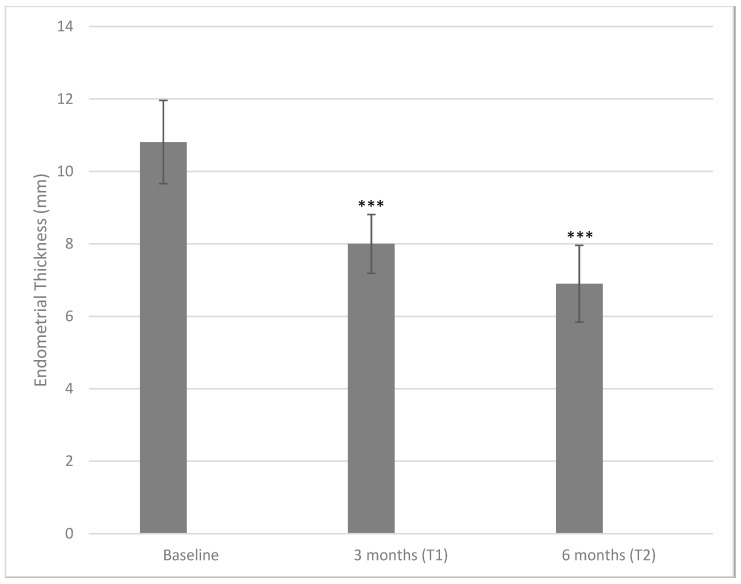
Values of endometrial thickness measured at baseline and after three and six months of treatment with 600 mg/die of D-chiro-inositol (*** *p* < 0.001 versus baseline). Endometrial thickness is reported as mean ± SD. A *p*-value ≤ 0.05 was considered statistically significant, significance T1 vs. T0 and T2 vs. T1.

Figure 1 depicts the effects of the treatment with DCI 600 mg per day taken by oral route for 3 and 6 months on the primary outcome, namely, the endometrial thickness. Following the treatment, the endometrial thickness decreased from a baseline value equal to 10.82 ± 1.15 mm to 8.00 ± 0.81 mm at three months (*p* < 0.001) and to 6.9 ± 1.06 mm at six months (*p* < 0.001, both when compared with baseline or three months). The average reduction corresponded to 26.06% from a baseline to a three-month time point and a further 13.75% from a three-month time point to a six-month time point, giving a total reduction of 36.23% from the baseline to a six-month time point.

**Figure 2:**
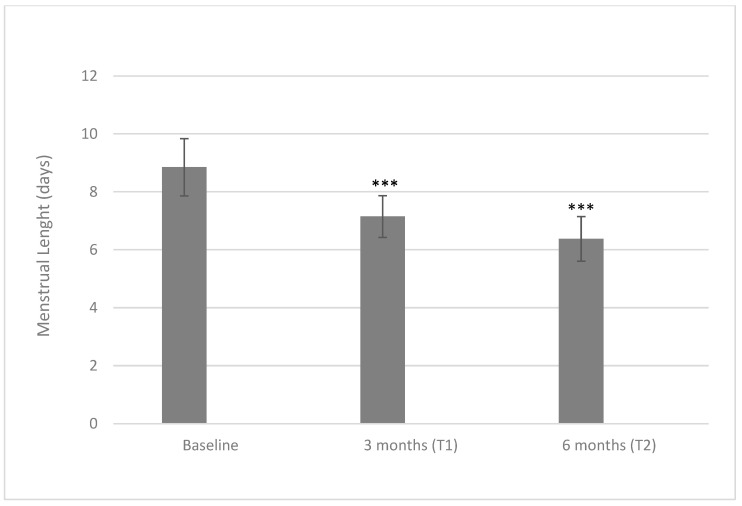
Values of menstrual length at baseline and after three and six months of treatment with 600 mg/die of D-chiro-inositol (*** *p* < 0.001 versus baseline). Menstrual length is reported as mean ± SD. A *p*-value ≤ 0.05 was considered statistically significant, significance T1 vs. T0 and T2 vs. T1.

When considering the menstrual length, the tested treatment based on a D-chiro-inositol therapy induced a reduction in the number of days of menstruation (Figure 2). At baseline, patients’ menstruations lasted for 8.85 ± 0.99 days, while at a three-month time point, the length fell to 7.15 ± 0.72 days (*p* < 0.001), reaching the minimum of 6.39 ± 0.77 days at a six-month time point (*p* < 0.001 both when compared with baseline or three months).

**Figure 3:**
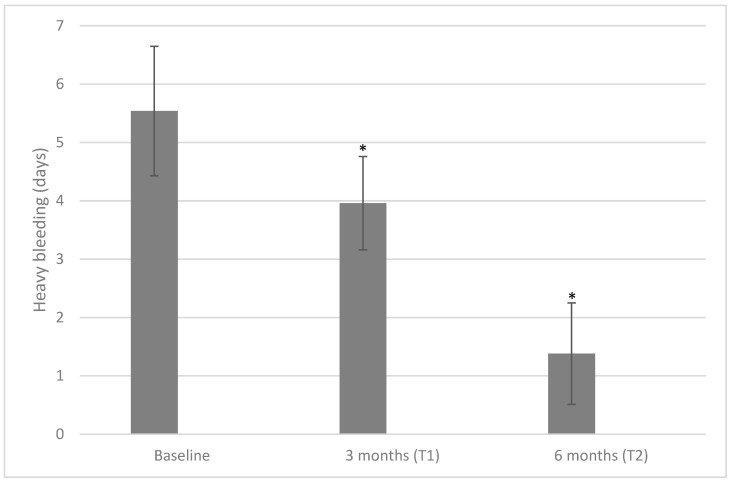
Values of heavy bleeding measured at baseline, T1, and T2 after treatment with 600 mg/die of D-chiro-inositol (* *p* < 0.05 versus baseline). Heavy bleeding is reported as mean ± SD. A *p*-value ≤ 0.05 was considered statistically significant, significance T1 vs. T0 and T2 vs. T1.

We also evaluated the number of days in which patients reported HMB (Figure 3). At baseline, the days with HMB were 5.54 ± 1.11, lowered to 3.96 ± 0.80 days after three months (*p* < 0.001) and further lowered to 1.38 ± 0.87 days after six months (*p* < 0.001 both when compared with a baseline or a three-month time point).

As our study reports multiple outcomes that significantly changed, we performed the Bonferroni–Holm method to assess the real extent of the significance. Table 2 reports the adjusted *p*-values and the relative significance. Our correction demonstrates that the independent values have a relevant significance even though we performed multiple analyses in the same study.

We also performed blood tests to check for changes in the hormones during the period of this study. We observed no significant variation in the levels of the hormones analyzed (Table 3).

## 3. Discussion

Unopposed estrogens may cause endometrial thickening by inducing the proliferation of endometrial cells. In addition, insulin stimulus may prompt the proliferation [3]. Based on the anti-estrogenic and insulin-sensitizing actions of D-chiro-inositol, we recommended a therapy based on this molecule to patients with AUB who were referred to our gynecological unit. We herein report the effect of such treatment, which induced a reduction in menstrual flow, heavy bleeding, and, most importantly, endometrial thickness. Indeed, endometrial thickening is the main result of unopposed estrogen stimulus, and it can be further supported by insulin signals despite patients having normal insulin levels [3].

The endometrial thickness is affected by hormonal changes depending on the phase of the cycle. The endometrium is thinner in the early post-menstrual period, growing gradually until it reaches its maximum thickness in the days before menstruation. In the proliferative phase, until the ovulation, the ovaries produce estrogens, thickening the endometrium due to the proliferation of cells [24]. Following ovulation, the dominant hormone is progesterone, produced by the corpus luteum. In the absence of pregnancy, the hormonal levels then decrease at the end of the secretive phase, leading to the degeneration and, thus, the thinning of the endometrial tissue. In the case of unopposed estrogens, this cycle shifts to a “more proliferative” process, resulting in abnormal endometrial thickness [24]. Although there are no reference cut-offs, the literature reports the upper limits of normal endometrial thickness in the pre-menopause state, depending on the days of the menstrual cycle [25,26]. Indeed, in our study, we measured the endometrial thickness between the 8th and the 10th days of the cycle. According to Park et al. [25], the cut-off value of thickness to define endometrium as abnormal is 8mm in the proliferative phase. Therefore, we included patients with thicker endometria, which appeared to have a higher risk of developing endometrial pathologies. The higher thickness of the endometrium is also associated with a higher chance of insurgence of the symptoms as HMB. HMB strongly affects the quality of women’s lives, especially in the perimenopausal period, heavily burdening the decision to take drugs, which may come with relevant side effects [2,27]. Such patients are recommended to take medications, generally progestogens, to counteract the proliferative phenomena.

Most of the guidelines for endometrial hyperplasia recommend the use of the levonorgestrel-based intra-uterine systems (IUS) as a first-line treatment in the absence of atypia [28]. According to these guidelines, the treatment with progestins is indicated in women who show a constant state of the disease after observation and in women with such symptoms as AUB. IUS is considered a first-line approach due to its efficiency, especially in the case of levonorgestrel-releasing intrauterine system (LNG-IUS). LNG-IUS is generally recommended as it has a higher rate of regression and fewer adverse effects than oral progestins [28]. However, progestogens may induce relevant undesired outcomes, including infertility, due to their contraceptive effects. Among the adverse effects that may derive from progestogen use, headache, abdominal pain, depressed mood, and a pre-menstrual syndrome-like condition are the most diffused [29,30].

To avoid the insurgence of side effects, we decided to test whether D-chiro-inositol could reduce the proliferation of the endometrium. D-chiro-inositol is one of the second messengers of insulin, and thus, its main clinical effect is to sensitize tissues to insulin signals. Over the years, DCI has been used in the clinical practice of several clinical pictures, including insulin resistance and PCOS [19,21,31]. During in vitro studies, DCI showed interesting properties, influencing the hormone production [20,23,32]. Combining such results with clinical studies, a dual role of DCI emerged; on the one hand, it had insulin-sensitizing properties, while on the other hand, it showed effects on the hormones [33,34]. The first insights into the role that D-chiro-inositol plays in hormonal production derive from the results of the studies from Nestler [35]. Recent evidence suggests that DCI can increase testosterone levels via the inhibition of the expression of aromatase [32]. The genic inhibition of aromatase allows the recovery of the physiological levels of hormones in the case of increased estrogens or reduced androgens. The literature suggests that this leads to the arrest or control of the progression of estrogen-sensitive pathologies, reducing the associated symptoms and improving the quality of life of women [3,17].

Several studies reported that aromatase is significantly overexpressed in estrogen-dependent pathologic tissues, as in endometriosis and endometrial hyperplasia, raising the local production of estrogen and enhancing the growth of lesions. Therefore, lowering estrogen levels represents a valid therapeutic strategy in the management of the pathologies characterized by unopposed estrogens, aromatase overexpression, and AUB [3].

Since endometrial hyperplasia represents the first step in the formation of endometrial cancer, patients reporting AUB or diagnosed with endometrial thickening should be carefully monitored. As previously mentioned, progestins represent the first-line pharmacological treatment, despite the non-negligible side effects [28]. However, up to 30% of the patients may experience the recurrence of the pathologies after complete regression. Furthermore, the optimal duration of progestin-based treatments is quite high and represents a problem for perimenopausal women who begin to experience metabolic and hormonal changes that become risk factors for standard hormonal therapies. For these reasons, several clinical trials aimed at demonstrating the effect of standard progestin-based therapies combined with other drugs similar to insulin sensitizers, such as metformin [12,13]. Indeed, a recent meta-analysis aimed at comparing the efficacy of metformin, either alone or associated with progestins, with the efficacy of progestogens in endometrial hyperplasia. The results suggest that metformin may be a useful treatment to counteract proliferation, also in combination with progestogens [12].

Recent evidence suggests that DCI has clinical effects comparable to metformin, inducing minor or null side effects. Therefore, DCI could be used in association with progestins to reduce the symptoms or the length of the pharmacological therapy [33]. In addition, DCI could represent an alternative to classic pharmacological therapies, especially if the patient has the desire for motherhood or is in perimenopause, when progestins may not be indicated [18,36]. Moreover, DCI is a safe molecule with almost no side effects; in fact, it appears in the list of GRAS molecules (Generally Recognized As Safe) published by FDA [10].

Herein we report a significant effect of DCI on reducing endometrial thickness and menstrual bleeding in perimenopausal women with endometrial thickening and AUB. This may derive from the dual action of DCI, especially DCI’s modulation of aromatase expression, which explains the important reduction in endometrial thickness and the improvement of the symptoms. Particularly, we report that DCI can interfere with the proliferative phenomena of thickened endometria. This is a result of primary importance, as this allows the possibility of introducing DCI-based therapies, preventing the patients from taking progestogen-based therapies. Moreover, we found out that DCI reduces the symptomatology associated with simple endometrial hyperplasia. This is probably due to the effectiveness of DCI in treating local proliferation, therefore removing the phenomenon at the base of the symptoms.

The strength of our study is the production of the first evidence of the effectiveness of D-chiro-inositol in an endometrial hyperplasia pathological context. Moreover, our data are valuable and encouraging, as the performed statistical correction grants the real significance of the data. Nonetheless, our study has some weaknesses. First of all, this is a pilot study involving only an intervention group, and therefore, such a study is missing appropriate controls. Indeed, the fact that this is a pilot study with a small sample size limits the magnitude of such effectiveness of D-chiro-inositol, calling for further demonstrations. Moreover, our study only involved patients with simple hyperplasia, limiting the potential application to such patients and excluding patients with atypical hyperplasia from the clinical target of D-chiro-inositol. Therefore, despite our promising results, these encouraging data need further confirmation through more structured studies. We encourage further randomized, double-blind, and placebo-controlled studies on larger samples to assess the actual effectiveness of DCI treatment.

## 4. Materials and Methods

This was a pilot study involving women with endometrial hyperplasia without atypia referring to the Women’s Health Centre and Alma Res Fertility Center between March and July 2022. This study’s design and all procedures were approved by the Ethical Committee of “Clinica Alma Res” (Unique Protocol ID 002/22). All women enrolled gave their oral informed consent after the explanation of this study’s purpose. This study was conducted following the Ethical Principles of the Helsinki Declaration and the national laws.

The inclusion criteria of this study were women aged at least 45, with endometrial thickness >8 mm on the 10th day of the menstrual cycle, with at least one associated symptom (HMB or long menses), and currently not under medical treatment. The exclusion criteria of this study were pregnancy or intention to become pregnant during the following six months, severe anemia, diabetes, hypertension, other medical morbidities, polycystic ovary syndrome, diagnosis of atypical endometrial hyperplasia, and current use of estrogen, progestin, oral contraceptives, corticosteroids, insulin, or food supplements containing D-chiro-inositol or other inositols.

Patients were treated orally with one tablet of D-chiro-inositol 600 mg and alfa-lactalbumin 60 μg (Tecadriol^®^ 600, Lo.Li. Pharma S.r.l., Rome, Italy) per day for 6 months. Patients were evaluated at baseline (T0), after three months of treatment (T1), and at the end of this study, after six months (T2). The primary outcome was the change in endometrial thickness analyzed by transvaginal ultrasonography (TVU). The secondary outcomes were the reduction in HMB, namely, the number of days with heavy bleeding and the length of menses. Transvaginal ultrasound was performed between the 8th and 10th day of the cycle in USL Umbria 2 Women’s Health Centre using the machine Voluson GE S8 (GE Healthcare, Chicago, IL, USA) by the same operator for all the patients. The day of the cycle in which the exam was performed on a single patient was the same in the three-time points. Diagnostic hysteroscopy to exclude the atypical form of endometrial hyperplasia was performed concomitantly in USL Umbria 2 Women’s Health Centre by the same operator for all the patients.

Statistical analysis was performed using paired *t*-test (2018 GraphPad Prism 8.0.1 Software, La Jolla, CA, USA); values are indicated as mean ± SD. We considered *p*-value ≤ 0.05 as statistically significant.

## 5. Conclusions

Endometrial hyperplasia is characterized by unopposed estrogens, and endometrial thickness is a result of the trophic push of high estrogen levels. The main symptom refers to a high volume of the menstrual period and a high length of menses that negatively impact the quality of life. In the present pilot study, DCI induced improvements in symptomatology without reporting any adverse events.

Thanks to the dual action of D-chiro-inositol, we tested for the first time such a molecule on women with endometrial thickening. The evidence herein provided suggests that DCI may represent a useful treatment in such gynecological diseases that often manifest with AUB. Nonetheless, this is a pilot study involving a small sample size and without any control group; therefore, our data should be validated in larger randomized and placebo-controlled trials. We encourage further research to prompt the use of DCI in such a clinical picture.

## Figures and Tables

**Table 1:** Anthropometric characteristics of patients at baseline and at the end of this study. Data are reported as mean ± standard deviation.

Parameter	Baseline	End of Study	*p*-Values
Height (m)	1.65 ± 0.07	1.65 ± 0.07	1
Weight (kg)	64.08 ± 5.35	63.77 ± 4.76	0.17
BMI (kg/m^2^)	23.63 ± 1.53	23.52 ± 1.39	0.18

**Table 2:** Original *p*-values and adjusted *p*-values calculated via Holm–Bonferroni method (* *p* < 0.05; ** *p* < 0.01).

Outcome	*p*-Value	Adjusted *p*-Value	Adjusted Significance
Endometrial thickness T0 versus T1	<0.001	0.006	**
Endometrial thickness T0 versus T2	<0.001	0.006	**
Days of heavy bleeding T0 versus T2	<0.001	0.007	**
Days of heavy bleeding T1 versus T2	<0.001	0.008	**
Menstruation length T0 versus T2	<0.001	0.010	*
Menstruation length T0 versus T1	<0.001	0.012	*
Endometrial thickness T1 versus T2	<0.001	0.016	*
Days of heavy bleeding T0 versus T1	<0.001	0.025	*
Menstruation length T1 versus T2	<0.001	0.049	*

**Table 3:** Levels of hormones at baseline and at the end of this study. Data are reported as mean ± standard deviation.

Hormone	Baseline	End of Study	*p*-Values
FSH (mUI/mL)	7.97 ± 1.36	8.07 ± 1.14	0.70
LH (mUI/mL)	12.56 ± 0.83	12.63 ± 0.84	0.61
FSH/LH	0.63 ± 0.10	0.64 ± 0.09	0.72
TSH (mcUI/mL)	2.59 ± 0.54	2.58 ± 0.40	0.87

## Data Availability

Data are available from the Corresponding Author on a reasonable request.
